# Response to: Query Design Logic in TriNetX Studies

**DOI:** 10.1002/hsr2.71690

**Published:** 2026-01-04

**Authors:** Robert J. Trager, Zachary A. Cupler, Jordan A. Gliedt, Ryan A. Fischer, Roshini Srinivasan, Hannah Thorfinnson

**Affiliations:** ^1^ Connor Whole Health University Hospitals Cleveland Medical Center Cleveland Ohio USA; ^2^ Department of Family Medicine and Community Health,School of Medicine Case Western Reserve University Cleveland Ohio USA; ^3^ Department of Biostatistics and Bioinformatics Clinical Research Training Program Duke University School of Medicine Durham North Carolina USA; ^4^ Physical Medicine & Rehabilitative Services Butler VA Health Care System Butler Pennsylvania USA; ^5^ Department of Neurosurgery Medical College of Wisconsin Milwaukee Wisconsin USA; ^6^ Section of General Internal Medicine, Department of Medicine Boston University Chobanian & Avedisian School of Medicine and Boston Medical Center Boston Massachusetts USA; ^7^ Duke University Department of Medicine Duke University Hospital Durham North Carolina USA; ^8^ Butler VA Medical Center Butler VA Health Care System Butler Pennsylvania USA

## 1 Introduction

We thank the author(s) for their interest in our study examining the association between spinal manipulative therapy (SMT) for low back pain (LBP) and opioid use disorder [1] and for their efforts to replicate our query. However, several key aspects of our design and inclusion logic were misinterpreted, leading to incorrect conclusions in their Letter.

## 2 Point‐by‐Point Response

### 2.1 Inclusion of Low Back Pain Patients

A central argument of the Letter that an “inclusion criterion for LBP was missing” from our methods is incorrect. As stated in our article, we included adults “receiving care for a new LBP episode with or without sciatica (ICD‐10: M54.4 or M54.5)” (Methods, section 2.2). Therefore, the suggestion that LBP diagnoses were not required is factually inaccurate and misrepresents our methods.

### 2.2 Misinterpretation of Eligibility Criteria and Index Date Alignment

The Letter misinterprets our handling of temporal logic and inclusion criteria. The authors suggest that we should have required “any LBP code before or on the same day as the patients′ index SMT event” or ibuprofen prescription. This does not align with our study′s objective or design. Our analysis focused specifically on new episodes of LBP, as stated in the abstract, methods, and supporting materials. We excluded prior LBP and radiculopathy episodes from −91 to −1 days before the index date (see Supporting Information: Table S1), ensuring that included patients were experiencing a new episode of LBP.

Illustratively, the Letter′s attempted replication query allowed SMT to occur “on or after the first instance” of LBP. This flexible logic could include patients with longstanding or recurrent LBP, testing a different clinical question altogether. Our approach, which required SMT to occur on the same date as the new episode of LBP diagnosis, ensured that the treatment corresponded to the index episode under study. Accordingly, the critique that some patients “were not diagnosed with LBP at study entry” is unfounded.

### 2.3 Failure to Apply Temporal Constraints

In attempting to reproduce our query, the author omitted the date range restrictions we imposed. Their supplemental file includes the phrase “the terms in this group occurred at any time,” whereas our study restricted inclusion to encounters between 2015 and 2023 (Abstract and section 2.1). Their omission could inflate cohort sizes and introduce older data that may not reflect contemporary opioid prescribing trends. In TriNetX, it is possible to obtain data from 20 years before the query date, such that their query likely captured data from 2005 to present, rather than 2015 onwards.

### 2.4 Database Growth Does Not Imply Invalidation

While the Letter correctly notes that the TriNetX network has expanded slightly since our analysis, it provides no evidence that this alters our study′s results or conclusions. For illustration, we re‐ran the cohort queries on October 30, 2025, obtaining slightly larger cohort sizes (30,202 patients). Expecting authors to continually repeat analyses following routine database expansion would be impractical and unnecessary. We agree, however, that if major changes in clinical practice or prescribing patterns occur, conducting an updated study would be appropriate.

## 3 Conclusions

In summary, the Letter′s critiques stem from misapplication of our design parameters and misrepresentation of our study purpose. The modest increase in cohort sizes is expected given growth in the TriNetX network size, and does not indicate flaws in our original work. We appreciate opportunities for post‐publication dialogue and welcome future critiques. However, we hope that further replication attempts accurately mirror the original study design before drawing conclusions about its accuracy.

## Author Contributions

**Robert J. Trager:** conceptualization, writing – original draft, writing – review and editing. **Zachary A. Cupler:** writing – review and editing. **Jordan A. Gliedt:** writing – review and editing. **Ryan A. Fischer:** writing – review and editing. **Roshini Srinivasan:** writing – review and editing. **Hannah Thorfinnson:** writing – review and editing.

## Disclosure

The views expressed are those of the authors and do not necessarily reflect the official policy or position of the US Department of Veterans Affairs or the US Government.

## Conflicts of Interest

Robert J. Trager received consulting fees from Merck for participation in an expert input meeting on chronic low back pain (October 2025) and received royalties from authoring two books on the topic of sciatica.
